# Readers of RNA Modification in Cancer and Their Anticancer Inhibitors

**DOI:** 10.3390/biom14070881

**Published:** 2024-07-22

**Authors:** Fengli Li, Wenjin Li

**Affiliations:** Institute for Advanced Study, Shenzhen University, Shenzhen 518060, China; 2200393009@email.szu.edu.cn

**Keywords:** readers, cancer, inhibitors, ncRNA, small molecule

## Abstract

Cancer treatment has always been a challenge for humanity. The inadequacies of current technologies underscore the limitations of our efforts against this disease. Nevertheless, the advent of targeted therapy has introduced a promising avenue, furnishing us with more efficacious tools. Consequently, researchers have turned their attention toward epigenetics, offering a novel perspective in this realm. The investigation of epigenetics has brought RNA readers to the forefront, as they play pivotal roles in recognizing and regulating RNA functions. Recently, the development of inhibitors targeting these RNA readers has emerged as a focal point in research and holds promise for further strides in targeted therapy. In this review, we comprehensively summarize various types of inhibitors targeting RNA readers, including non-coding RNA (ncRNA) inhibitors, small-molecule inhibitors, and other potential inhibitors. We systematically elucidate their mechanisms in suppressing cancer progression by inhibiting readers, aiming to present inhibitors of readers at the current stage and provide more insights into the development of anticancer drugs.

## 1. Introduction

Tumors, characterized by abnormal and excessive growth of tissues, present a complex challenge in medical oncology. Benign tumors typically exhibit slow growth rates and lack the ability to invade adjacent tissues or metastasize to distant sites. However, the transformation of a benign tumor into cancer heralds a host of formidable challenges, including metastasis, recurrence, treatment resistance, and tumor heterogeneity. Despite significant strides in cancer therapeutics, there remains a substantial gap in effective treatment strategies. Targeted therapy has emerged as a promising approach that focuses on specific oncogenes or proteins expressed in cancer cells. These targeted agents act by modulating signaling pathways or molecular targets crucial for cancer cell survival and proliferation. Compared to conventional chemotherapy, targeted therapy offers improved selectivity, reduced side effects, and enhanced tolerability. Therefore, the development of novel anticancer drugs with targeted mechanisms has become a pivotal pursuit in cancer research [1,2,3].

RNA modification refers to the chemical alteration of RNA molecules without affecting the underlying genome. It plays a pivotal role in the epigenetic regulation of transcriptional processes, with 334 types of RNA modifications identified across various organisms [4]. Common RNA modifications in humans include 5-Methylcytosine (m5C), N6-methyladenosine (m6A), N1-methyladenosine, and 7-Methylguanosine [5]. RNA modification significantly influences RNA metabolism processes, such as gene transcription, translation, stability, splicing, and nuclear localization [6]. These modifications are dynamically and reversibly regulated by regulators, including methyltransferases (writers), demethylases (erasers), and RNA modification-binding proteins (readers) [7]. Writers deposit RNA modifications, erasers remove RNA modifications from target genes, and readers perform their functions by recognizing the RNA modification sites in target genes [8]. The impact of RNA modification on RNA metabolism largely relies on the recognition by various RNA modification-binding proteins (readers), which include the YTH domain family, heterogeneous nuclear ribonucleoproteins (HNRNPs), and insulin-like growth factor 2 mRNA-binding proteins (IGF2BPs) [9]. Readers not only participate in multiple RNA metabolism processes but also play crucial roles in various biological processes, such as tumorigenesis, hematopoiesis, viral replication, immune response, and lipid metabolism, contributing to their significant involvement in cellular functions [9]. Dysregulation of readers, frequently observed as upregulation, has been identified as a significant hallmark of many cancers and is often associated with poor prognosis in patients with cancer. For instance, elevated levels of YTHDF1 and YTHDC2 are adverse prognostic factors for gastric cancer (GC) [10,11], while increased IGF2BP3 levels are adverse prognostic factors for bladder cancer [12]. Hence, targeting readers for cancer therapy development holds significant promise.

Indeed, numerous research groups have engaged in the development of inhibitors targeting RNA modification-related enzymes and have achieved significant progress. Typical examples are the METTL family [13], whose members belong to the writer proteins, of which the relatively well-characterized member is METTL3, which leads the pack in terms of the number of its inhibitors [14]. Regarding erasers, the number of identified inhibitors is slightly inferior to that of the writer proteins, but within the ALKBH family, FTO has a wide range of inhibitors [15], including natural products [16], manually designed inhibitors [17], and FDA-approved drugs [18]. The mechanisms of action of these inhibitors in various diseases are gradually being revealed. Compared to writers and erasers, the understanding of readers has only become relatively comprehensive in recent years, and researchers have recognized their immense research value. Given the expanding roles of reader proteins in various pathologies, the development of their inhibitors has also received increasing attention in the research field. Both the number of reader proteins and the overall number of their inhibitors are higher than those of the other two types of enzymes. Moreover, recent research findings and the growing number of research teams suggest that the development of reader inhibitors as anticancer drugs still holds limitless potential and possibilities.

Notably, several small-scale review articles focusing on reader inhibitors, such as human antigen R (HuR) and HNRNPA1, have been published [19,20,21]. Here, firstly, we provide an overview of the primary regulatory functions and mechanisms of action of RNA readers as critical regulators, as well as the various mechanisms by which dysregulation of readers in signaling pathways contributes to cancer development. Understanding these aspects is a prerequisite for developing various inhibitors. Subsequently, our focus shifts to a comprehensive review of non-coding RNA (ncRNA) inhibitors, which have yet to be summarized extensively, as well as small-molecule inhibitors and other potential inhibitors. This comprehensive review aims to facilitate further clinical drug screening and offer diverse approaches for anticancer drug development.

## 2. Readers of RNA Modification

As of today, the only readers found in humans are those corresponding to m5C, m6A, and N6-methyladenosine with 2′-O-methylation (m6Am).

### 2.1. Readers of m5C

m5C is a common form of RNA modification, which refers to the addition of a methyl group to the fifth carbon atom of the cytosine base in RNA molecules [22,23]. The biological roles of this modification in RNA include translation, degradation, splicing, export, and folding [24]. Currently, readers of m5C primarily include the Aly/REF export factor (ALYREF) and Y-box binding protein 1 (YBX-1) in humans. ALYREF is mainly localized to the nucleus and is an essential component of the transcription export complex (TREX). It can specifically recognize and bind to m5C-modified mRNAs, mediating their transportation between the nucleus and cytoplasm [25,26,27]. ALYREF binds to a region near the 5′ end of the mRNA in a CBP80-dependent manner and PABPN1-dependent manner binding near the 3′ end of the mRNA. Furthermore, the 3′ processing factor CstF64 directly interacts with ALYREF for the overall binding of ALYREF on the mRNA. ALYREF promotes the nuclear export of intronless mRNA through the aforementioned binding sites [28]. YBX-1 is a member of the family of DNA- and RNA-binding proteins with an evolutionarily ancient and conserved cold-shock domain [29]. As a ribonucleoprotein, YBX-1 has two important states: phosphorylation and dephosphorylation. Non-phosphorylated YBX-1 binds to messenger ribonucleoprotein particles that inhibit internal ribosome-dependent mRNA translation. Once phosphorylated at S102, its mRNA-binding ability is weakened, permitting the translation of mRNAs. Also, phosphorylation of YBX-1 allows its translocation to the nucleus with an increase in YBX-1-related gene expression [30].

### 2.2. Readers of m6A

m6A is a modification in which a methyl group is added to the sixth nitrogen atom of the adenine base in RNA molecules. It can occur in various types of RNA, including mRNA, tRNA, rRNA, microRNA (miRNA), and long non-coding RNA (lncRNA), with mRNA being particularly prominent. On average, there are 3–5 m6A sites in each mRNA molecule [31]. Among the known RNA modifications, m6A modification has been extensively studied due to its high abundance in eukaryotic cells [32]. Functionally, m6A is involved in nearly all RNA metabolic processes, including translation, degradation, splicing, export, and folding [33]. The most common human m6A readers include 17 members. Different readers have distinct m6A localization functions. For example, HNRNPC, HNRNPA2B1, and HNRNPG are nuclear m6A readers, while YTHDF1/2/3 and IGF2BP1/2/3 are typical cytoplasmic RNA readers for m6A modification [34]. 

HNRNPs are structures that package heterogeneous nuclear RNA and are among the main components of the cell nucleus [35]. The HNRNPs family from A1 to U, comprising approximately 20 major peptides [36], includes HNRNPA1, HNRNPA2B1, HNRNPC, and HNRNPG, which have been found to possess the ability to recognize RNA m6A. HNRNPA1 was initially identified as one of the core proteins of ribonucleoprotein complexes [21,37]. Acting as a potent splicing factor, HNRNPA1 regulates multiple splicing events by binding to splicing sites and regulatory elements in exons or introns and cooperating with other splicing factors or antagonists [38]. In transcription, HNRNPA1 can regulate gene transcription by interacting directly with promoters or indirectly by influencing Pol II activity [39]. In translation, HNRNPA1 can affect mRNA translation by interacting with internal ribosome entry sites (IRES) on mRNA [40]. Additionally, HNRNPA1 can alter mRNA stability by binding to specific positions in the 3′-UTR, participating in miRNA biogenesis and nonsense-mediated mRNA decay (NMD) pathways [21,41]. HNRNPC is another abundant nuclear reader responsible for pre-mRNA processing. Liu et al. discovered a mechanism called ‘the m(6)A-switch’, where m6A modifies the local structure of mRNA and lncRNA, facilitating HNRNPC binding [42]. HNRNPA2B1 and HNRNPG participate in the splicing of m6A-tagged mRNA [43,44]. Moreover, HNRNPA2B1 binds to m6A in a subset of primary miRNA transcripts and promotes primary miRNA processing [43].

In addition to members of the HNRNPs family, another major class of m6A readers in humans is the YTH domain family, which includes YTHDF1, YTHDF2, YTHDF3, YTHDC1, and YTHDC2. The functions of YTHDF1 primarily involve promoting target RNA translation and stabilizing RNA [45]. Regarding translation promotion, YTHDF1 triggers translation initiation through eukaryotic initiation factor 3. YTHDF1 may also be responsible for m6A-induced Snail mRNA translation elongation by interacting with the elongation factor eEF-2 in cancer cells [46]. Furthermore, YTHDF1 can promote translation in a cap-dependent or cap-independent manner, with cap-independent methods requiring IRES [47]. YTHDF2 was the first discovered m6A-binding reader. It promotes mRNA degradation by recruiting the CCR4-NOT deadenylase complex [48,49]. Specifically, after YTHDF 2 binds methylated mRNA through the YTH domain of the protein, multiple sites in the N-terminal region are involved in the recruitment of the CCR4-NOT complex, which mediates the degradation of the polyadenine tail of mRNA and triggers de-adenylation-dependent mRNA decay [50]. YTHDF3, besides regulating m6A-mRNA stability and translation [51], recruits eIF4G2 to m6A sites, driving the translation initiation of circular RNA (circRNAs) [52]. Often, there are complex interactions among YTHDF1/2/3. For example, YTHDF3 can act as a modulator for the other two, cooperating with YTHDF1 to bind m6A-mRNA and interact with 40S and 60S ribosomal subunits to promote mRNA translation while interacting with YTHDF2 to affect the decay of methylated mRNA transcripts [53].

In eukaryotic cells, precursor mRNA splicing into mature mRNA is a crucial step for gene expression and protein diversity [54]. Serine/arginine-rich splicing factors (SRSF) are important proteins in the cell nucleus that regulate the splicing process by binding to mRNA precursors. YTHDC1 is the only YTH domain protein located in the nuclear speckles of mammalian somatic cells [55]. It directly regulates pre-mRNA splicing by binding to members of the SRSF family. For example, YTHDC1 recruits SRSF3 while blocking the binding of SRSF10 to its target RNA, directly regulating splicing events [56]. YTHDC1 can also interact with the pre-mRNA 3′ end processing factors SRSF3 and SRSF7, which is reflected in oocyte growth and maturation [57]. The interaction between YTHDC1 and SRSF3 can also transport mRNA to the nuclear RNA export factor for transport from the nucleus to the cytoplasm [58]. YTHDC2, as an m6A reader, is the only ATPase with RNA-induced 3′–5′ RNA helicase activity. This makes it essential for successful meiotic gene expression programs in mammalian germ cells and essential for both male and female fertility [59]. Since YTHDC2 has an RNA helicase domain, it can promote the translation of structured mRNAs by unwinding their secondary structure [60].

IGF2BPs, including IGF2BP1/2/3, form a highly conserved m6A reader family that targets thousands of mRNA transcripts by recognizing the consensus GG(m6A)C sequence [61]. IGF2BPs are typically enriched in the cytoplasm and form ribonucleoprotein granules near the nucleus [62]. The IGF2BPs family consists of two RNA recognition motif (RRM) domains and four K homology (KH) domains [62,63,64]. Phosphorylation of the connecting region between KH 2 and 3 of IGF2BP1 activates β-actin mRNA translation in a Src-dependent manner and induces degradation of cytoplasmic messenger ribonucleoprotein granules [65]. Simultaneously, IGF2BP1 can recruit human antigen R (HuR) to prevent m6A-containing mRNA degradation and promote its translation [61]. Phosphorylation by mTOR at the junction of RRM2 and KH1 in IGF2BP2 promotes its binding to IGF precursor sequences and promotes the translation initiation of IGF mRNA by entering the IRES [66]. Both the RRM1 and RRM2 domains in IGF2BP3 form complexes with RNA, but only RRM1 participates in RNA binding and recognizes dinucleotide sequences [67,68].

Among others, one of the most well-characterized m6A readers is HuR, the protein product of the embryonic lethal and abnormal vision gene *ELAVL1* [19]. Under normal physiological conditions, HuR primarily resides in the nucleus. However, upon exposure to internal and/or external stresses, HuR can translocate to the cytoplasm, where it stabilizes and enhances the translation of target mRNAs [69]. Specifically, HuR contains three RRMs, RRM1–3, with a hinge region between RRM2 and RRM3 that contains the HuR nucleocytoplasmic shuttling domain. HuR recognizes and binds to the 3′UTR region of mRNA modified with m6A within the cell nucleus and subsequently escorts them through the nuclear pores [70,71,72]. Fragile X mental retardation protein (FMRP) is also an m6A-modified reader, and the lack of FMRP in neurons results in cognitive impairment [73]. FMRP contains two RNA-binding domains (KH1 and KH2) and a C-terminal RG-rich region involved in RNA binding. It regulates RNA alternative splicing, mRNA stability, and translation by binding to target mRNA-coding regions or 3′UTRs. FMRP also participates in RNA transport and other critical biological processes [74,75]. 

The reader protein leucine-rich pentatricopeptide repeat (PPR) containing (LRPPRC), contains multiple copies of the leucine-rich nuclear translocation signal at the N-terminus, and the C-terminal sequence contains multiple PPR motifs for RNA binding [76,77]. LRPPRC associates with the poly(A) mRNA binding of HNRNPA1 and participates in its nuclear mRNA maturation and export [78]. Little is known about the biological functions of LRPPRC, and further research is needed to understand its role better. Proline-rich coiled-coil 2A (PRRC2A) is a newly discovered reader that primarily binds to the shared sequence UGGAC in m6A-modified transcripts. It competes with YTHDF2 in a post-transcriptional m6A-dependent manner, regulating the stability of *Olig2* mRNA in the mouse neuroepithelium [79]. eIF3 is a multi-protein complex, and under stress or disease conditions where eIF4E (a typical cap-binding protein) is inhibited, it can bind to m6A-modified mRNA in the 5′-UTR and recruit ribosomal subunits to initiate translation [46,80].

### 2.3. Readers of m6Am

The biological synthesis of mRNA involves adding an N7-methylguanosine cap with a triphosphate linkage at the 5′ end of the mRNA to protect the transcript from rapid nucleolytic degradation [81,82]. If the first nucleotide after the m7G cap is 2′-O-methyladenosine (Am), it can undergo further methylation at the N6 position by an unidentified nuclear methyltransferase 9 to form N6,2′-O-dimethyladenosine (m6Am) [83]. m6Am is a reversible modification that affects the fate of cellular mRNA, with transcripts starting with m6Am being significantly more stable than those starting with other nucleotides [84]. So far, the only identified reader of m6Am in eukaryotic cells is decapping mRNA 2 (DCP2), a major decapping enzyme in 5′–3′ mRNA degradation. DCP2 consists of an N-terminal regulatory domain, a catalytic domain, and an intrinsically disordered C-terminal tail. DCP2 catalyzes the hydrolysis of the 5′ cap to release m7GDP and 5′ monophosphate RNA, which can then be degraded by conserved 5′–3′ exonucleases [85,86]. Research by Jan Mauer et al. has shown that m6Am confers resistance to DCP2, inhibiting decapping and stabilizing cellular mRNA transcripts [86,87]. Clarification of the primary function makes it easier to determine the role of readers in the cancer pathway and the development of targeted inhibitors.

## 3. The Oncogenic Mechanism of Readers

In cancer, the behavior of different readers varies significantly. Changes in their own levels, either upregulation or downregulation, can exert oncogenic or tumor-suppressive effects. For example, in the development of human CRC, YTHDC2 overexpression is associated with cancer cell migration [88]. However, in the treatment of nasopharyngeal carcinoma, high levels of YTHDC2 will lead to radiotherapy resistance [89]. Additionally, they can promote or inhibit cancer by recognizing RNA modifications to facilitate the translation or degradation of target RNAs. The pro-oncogenic effects of readers are the most widely recognized, and inhibition of readers as pro-oncogenic factors is the most understandable and feasible from the point of view of discovering therapeutic agents for cancer treatment. Here, we primarily focus on discussing a portion of the mechanisms by which readers exert carcinogenic effects. 

Recent observations indicate that ALYREF regulates gene expression in various cancers, participating in tumorigenesis and progression by promoting malignant proliferation, invasion, and metastasis and inhibiting the apoptosis of tumor cells [90,91]. High expression of ALYREF correlates with poor prognosis in patients, as evidenced in hepatocellular carcinoma (HCC), glioblastoma, glioma, neuroblastoma, lung adenocarcinoma, bladder cancer, and breast cancer [92]. Klec et al. found that ALYREF can promote breast cancer by binding to the Nuclear Enriched Abundant Transcript 1(*NEAT1*) promoter region, enhancing the overall transcriptional activity of *NEAT 1* and stabilizing Cleavage and Polyadenylation Specific Factor 6 [93]. ALYREF is significantly upregulated in urothelial bladder carcinoma (UCB) and promotes the splicing and stability of highly methylated RAB, member RAS oncogene family-like 6, and thymidine kinase 1 mRNA in an m5C-dependent manner, enhancing UCB cell proliferation and invasion [94]. 

YBX-1 exhibits high expression levels in various tumors, including breast cancer, prostate cancer, osteosarcoma, lung cancer, colorectal cancer (CRC), glioblastoma, ovarian cancer (OC), GC, and melanoma. Its overexpression is widely recognized as a hallmark of cancer [95]. YBX-1 is overexpressed in both subsets of pediatric glioblastoma but not in adult glioblastoma. In one subset, Faury et al. hypothesized that Akt-mediated phosphorylation of YBX-1 contributes to gliomagenesis in pediatric glioblastoma by attenuating the translational repression of many pro-mRNAs by YBX-1, increasing the level of epidermal growth factor receptor, and interfering with the function of p53, A second subset, which is not associated with activation of Akt and Ras pathways, and which may originate from astrocyte progenitor cells [96]. Based on a clinical database, Jiang et al. demonstrated that Kruppel-like factor 5 (KLF5) is positively correlated with YBX-1 expression in breast cancer patients. Mechanistically, YBX-1 enhances KLF5 expression through transcriptional activation and stabilizes KLF5 in a manner that is dependent on RNA m5C modification. In addition, phosphorylation of YBX-1 Ser102 promotes the formation of the YBX-1/KLF5 transcriptional complex, which jointly regulates the expression of *keratin 16* and *lymphocyte antigen 6 family member D* and promotes the proliferation of basal-like breast cancer cells [97]. 

YTHDF1 is highly expressed in GC tissues and is associated with poor prognosis in patients with GC. It is an independent prognostic factor for poor prognosis in patients with GC. Chen et al. found that ubiquitin-specific protease 14 mRNA is an m6A modification target of YTHDF1 in GC cells [98]. Ubiquitin-specific protease 14 enhances cisplatin resistance and accelerates cell proliferation and migration in GC by affecting the Akt/ERK signaling pathway [11]. YTHDF2 expression is upregulated at both the mRNA and protein levels in pancreatic cancer tissues, and its expression is higher in late-stage clinical patients with pancreatic cancer. Knockdown of YTHDF2 can downregulate p-Akt expression and inhibit the Akt/GSK 3 β/CyclinD 1 signaling pathway, thus inhibiting cancer cell proliferation. However, Chen et al. found a “migration-proliferation dichotomy” phenomenon of YTHDF2 in pancreatic cancer, which promotes pancreatic cancer cell proliferation but inhibits migration and invasion. Therefore, YTHDF2 as a target for pancreatic cancer still needs serious consideration [99]. High expression of YTHDF3 is associated with poor disease-free survival and overall survival in triple-negative breast cancer patients. ZEB1 is a major element in the transcription factor network controlling epithelial-to-mesenchymal transition (EMT) [100]. YTHDF3 can enhance ZEB1 mRNA stability in an m6A-dependent manner, thereby promoting the migration, invasion, and metastasis of triple-negative breast cancer [101]. 

YTHDC1 typically exhibits inhibitory effects on cancer. YTHDC2 is significantly upregulated in human GC tissues and is associated with poor prognosis. Yes-Associated Protein (YAP) is an important transcriptional co-activator, and its abnormal activation is associated with tumor occurrence, development, and progression. Yuan et al. found that YTHDC2 can recognize m6A-modified YAP mRNA at the 5′-UTR, thereby enhancing YAP translation efficiency without affecting its mRNA level. Conversely, YAP/TEAD directly targets the −843∼−831 region of the YTHDC2 promoter, activating YTHDC2 transcription and forming a positive regulatory loop [10]. 

The SRF/IGF2BP1-, miRNome-, and m6A-dependent gene expression control is a conserved oncogenic driver network in cancer [102]. IGF2BP1 promotes SRF expression in a conservative m6A-dependent manner by impairing the decay of SRF mRNA guided by miRNA, thereby promoting the expression of genes such as *PDLIM7* and *FOXK1* that can promote tumor cell growth, ultimately promoting tumor cell growth and invasion [102]. Gene mutations of ErbB2 have been confirmed in various types of cancers. IGF2BP2 can promote ErbB2 expression, thereby enhancing CRC cell proliferation, invasion, and migration and inhibiting cell apoptosis. Mechanistically, IGF2BP2 recognizes m6A on YAP mRNA to promote its mRNA translation, and then YAP upregulates ErbB2 expression by promoting the enrichment of TEA Domain Transcription Factor 4 in the ErbB2 promoter region [103]. IGF2BP3 is overexpressed in bladder cancer tissue, and its high expression is closely related to poor prognosis in patients with bladder cancer. Specifically, IGF2BP3 promotes the activation of the JAK/STAT pathway in bladder cancer cells, thereby promoting their proliferation [12]. 

HuR stabilizes CKLF-like MARVEL transmembrane domain-containing protein 6 (CMTM6) by directly binding to the AU-rich elements in its 3′UTR and upregulating its mRNA expression. CMTM6 is a key factor controlling the stability of cell surface PD-L1 and consequently contributes to the immune evasion of tumor cells. This suggests the potential of combining HuR inhibitors with PD-1/PD-L1 antibodies for cancer immunotherapy [104]. The new m6A reader FMR1 is also involved in the regulation of cancer progression. In CRC, FMR1 can recognize m6A modification sites in the mRNA of key molecules involved in cancer occurrence and targeted therapy, such as epidermal growth factor receptor, and maintain their stability and expression in an m6A-dependent manner, thereby promoting tumorigenesis and metastasis of CRC [74]. 

**Figure 1:**
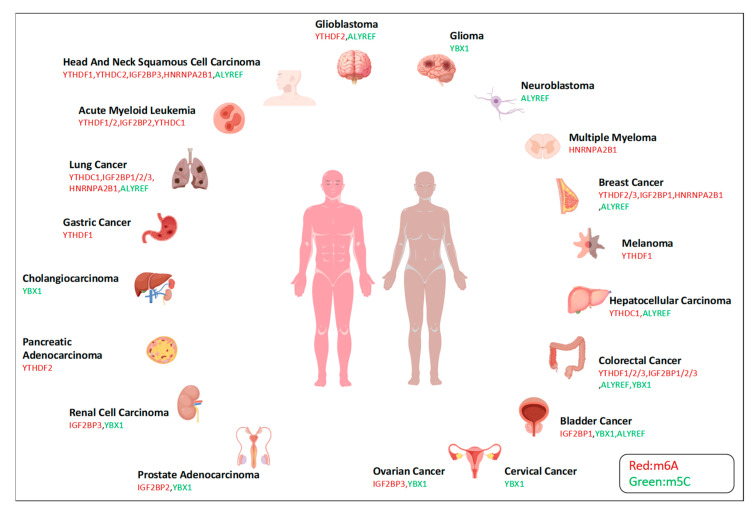
The role of readers in human cancers.

HNRNPA1 increases the expression of cyclin D1 by directly binding to the 3′UTR of Vaccinia-related kinase 1 (VRK1) mRNA, thereby positively regulating the translation of *VRK1* [105]. HNRNPC can bind to and stabilize WDR77 mRNA. WDR77 sequentially promotes the G1/S phase transition of the cell cycle and promotes the proliferation of breast cancer cells [106]. Reactive oxygen species (ROS) are mainly produced by the mitochondria and have been shown to play an important role in stress signal transduction in cancer cells. LRPPRC regulates ROS balance through mt-mRNA metabolism and the circANKHD1/FOXM1 axis, protecting UCB cells from oxidative stress [107]. The role played by dysregulation of readers in cancer has been summarized in detail in many reviews and is shown in Figure 1 [9,92,108,109,110].

## 4. Non-Coding RNA Inhibitors of Readers in Cancer

ncRNA is RNA transcribed from the genome, which was once thought to be meaningless by-products of transcription because they do not translate into proteins. However, the evolution of organisms dictates that every “worker” has its own function. With further research, it has been discovered that these ncRNAs are involved in many important biological processes [111]. ncRNAs mainly include miRNA, lncRNA, and circRNA [112]. RNA epigenetic regulators can modulate the modification of ncRNA, as mentioned earlier. Additionally, ncRNAs can act as inhibitors to regulate gene expression by acting on these regulators, which, in turn, achieve anticancer effects. Here, we summarize three types of non-coding RNAs, i.e., miRNA, lncRNA, and cicrRNA, as reader inhibitors in different cancers. Additionally, we discuss the novel development of siRNA nanoencapsulation as a potential inhibitor, which is emerging as a promising approach.

### 4.1. miRNA

miRNAs belong to a class of small ncRNAs originating from primary transcripts (pri-miRNAs) transcribed by RNA polymerase II/III, producing endogenous short RNAs (~22 nt) [113]. Functionally, miRNAs target the mRNA 3′UTR through complementary base pairing, driving interference with mRNA translation and inducing mRNA degradation. miRNAs reflect their interference with cancer-related mRNA, demonstrating their impact on cancer stem cell differentiation, proliferation, metastasis, prognosis, and therapeutic value across various cancers over the past few decades [114].

A single miRNA can play a significant role in tumorigenesis and progression. MiR-382, located on chromosome 14q32, has been demonstrated to participate in the development, metastasis, and therapy resistance of various cancers [115]. Xu et al. identified *YBX-1* as a target gene of miR-382 through algorithms predicting the mRNA targets of miRNAs. Overexpression of miR-382 inhibits EMT and metastasis by suppressing YBX-1 expression. In vivo experiments have demonstrated that miR-382 overexpression inhibits tumorigenesis and prevents disease recurrence in osteosarcoma patients when combined with doxorubicin [116]. Additionally, Wang et al. found that miR-382-5p (5′ end fragment of miR-382) negatively regulates the expression of YBX-1 when transfected into U251 and U87 glioma cells [117]. The non-translated region of *YBX-1* linked to a luciferase reporter gene demonstrates direct binding of miR-137 to YBX-1, suppressing its expression and inhibiting Malignant Pleural Mesothelioma cell growth and colony formation [118]. Furthermore, the knockdown of miR-137 increases CRC cell resistance to oxaliplatin (OXA). YBX-1 is a direct target gene of miR-137 in CRC cells, and miR-137 targeting of YBX-1 expression may be a potential strategy to overcome OXA resistance in human CRC [119]. Li et al. demonstrated that inhibition of YBX-1 by miR-216a suppresses the proliferation and invasion of diffuse large B-cell lymphoma [120]. 

HuR, as a crucial target for cancer therapy, has been confirmed to be regulated by miRNAs in some reports. Upregulation of miR-324-5p can inhibit colon cancer cell proliferation and invasion by targeting HuR [121]. In 2010, miR-16 regulation of the HuR translation pathway was first linked to human breast cancer [122]. Subsequently, it was found that miRNA-125a can inhibit breast cancer cell growth by targeting HuR [123]. Diabetic retinopathy is a common complication of diabetes, characterized by microaneurysms and irregular blood vessels, leading to visual impairment. MiR-192-5p can directly target HuR, destabilizing phosphatidylinositol 3-kinase δ mRNA and inhibiting the proliferation, migration, and angiogenesis of microvascular endothelial cells [124]. 

Numerous miRNA inhibitors have been discovered targeting HNRNPA1 in various cancers. The Warburg effect is a distinct metabolic phenomenon observed in cancer cells, where they preferentially metabolize glucose via glycolysis, even in the presence of oxygen, rather than oxidative phosphorylation [125]. Pyruvate kinase (PKM) is a key rate-limiting enzyme in glycolysis, with PKM alternatively spliced into M1 (PKM1) or M2 (PKM2) isoforms [126]. Only PKM2 is expressed in cancer cells to promote glycolysis, while PKM1 is expressed in normal differentiated tissues, promoting oxidative phosphorylation instead of glycolysis [127]. Converting PKM expression from the PKM2 isoform to the PKM1 isoform inhibits the Warburg effect and cancer cell growth [128]. PKM1 and PKM2 are generated by mutually exclusive splicing of the PKM gene, primarily mediated by the alternative splicing proteins PTB1, HNRNPA1, and HNRNPA2 [129]. Targeted regulation of these three splicing proteins theoretically achieves the transition from PKM2 to PKM1, thereby reversing the Warburg effect. Indeed, studies have utilized miR-137 and miR-206 overexpression to target HNRNPA1 and regulate PKM alternative splicing, inhibit PKM2 expression, and attenuate the Warburg effect and proliferation of CRC cells directly [130,131]. 

miR-18a induces apoptosis in colon cancer cells by directly binding to oncogenic HNRNPA1. Additionally, in this study, it was found that the complex formed by miR-18a and HNRNPA1 was degraded via the autophagolysosomal pathway, marking the first report demonstrating a new function of miRNAs in inhibiting cancer progression by forming a complex with an RNA-binding protein degraded in the autophagolysosomal pathway [132]. Moreover, miR-135a-5p and miR-149-5p targets regulate HNRNPA1 expression in renal cancer [133]. miR-490 targets hnRNPA to inhibit GC [134]. Upregulation of miR-424 and miR-503 can inhibit the upregulation of HNRNPA1 in breast cancer [135]. miR-26a and miR-584 inhibit the binding of HNRNPA1-CDK6 mRNA and induce apoptosis in CRC cells [136]. MiR-582-5p inhibits the proliferation and apoptosis of chronic lymphocytic leukemia cells by downregulating HNRNPA1 and upregulating IκBα expression, thereby suppressing NF-κB activity [137].

OC is the deadliest cancer in women [138]. miR-130c-135p inhibits OC progression and reduces m6A levels by regulating CDK19 mRNA stability through targeted inhibition of HNRNPA2B1 [139]. Another highly expressed m6A reader found in OC is HNRNPC. Elevated expression of miR-744-5p directly downregulates the mRNA and protein expression of nuclear factor IX (NFIX) and HNRNPC. HNRNPC leads to reduced expression of miR-21 and decreased Akt phosphorylation, while NFIX reduces Bcl-2 levels, resulting in detectable pro-apoptotic effects. Notably, miR-744-5p overexpression, along with cisplatin treatment, leads to cumulative pro-apoptotic effects [140]. Increased expression of hepatocyte nuclear factor 4 γ (HNF4G) and IGF2BP2 was observed in lung cancer tissues collected from patients. IGF2BP2 recognizes m6A to enhance the expression of thymidine kinase 1 (TK1), promoting angiogenesis. Computer simulation analysis identified HNF4G as a target of miR-320b. Knocking down HNF4G with miR-320b inhibits IGF2BP2 expression, suppressing cancer cell invasion and tube formation [141]. IGF2BP2 enhances TK1 mRNA stability by recognizing its m6A modification to enhance its expression, subsequently promoting the development of esophageal squamous cell carcinoma. Overexpression of miR-200b reverses this effect [142]. MiR-216b inhibits liver cancer cell proliferation, migration, and invasion by regulating IGF2BP2 and is modulated by HBx [143]. In gallbladder carcinoma, let-7g-5 p is an inhibitor of IGF 2BP 3, which binds directly to KLK 5 mRNA, and inhibition of KLK 5 reduces PAR2 expression and downregulates phosphorylated Akt expression [144].

Mutation of the P53 gene leads to chemotherapy resistance in CRC. Yang et al. confirmed that LRPPRC is a key downstream factor and therapeutic target induced by P53 mutation-mediated chemotherapy resistance. The accumulation of multidrug resistance 1 (MDR1) promotes drug resistance. Wild-type P53 negatively regulates LRPPRC, affecting the stability of MDR1 mRNA, while mutated P53 fails to suppress LRPPRC after DNA damage, leading to increased MDR1 transcription. Another key regulatory factor in this axis is miR-34a. LRPPRC is the direct target of miR-34a, and promoting the expression of miR-34a can reduce LRPPRC protein levels, thereby potentially disrupting chemoresistance in CRC [145].

Endothelial cells (ECs) can transport different types of biomolecules, such as proteins, mRNAs, and miRNAs, through the secretion of extracellular vesicles (EVs), thereby enabling intercellular signaling [146]. Among them, the typical miR-376c can be loaded into EVs and acts on non-small cell lung cancer (NSCLC). Specifically, ECs import miR-376c into NSCLC cells via EVs, which then target intracellular YTHDF1 and inhibit its expression, thereby disrupting the Wnt/β-catenin pathway mediated by YTHDF1 and ultimately leading to the inhibition of cell proliferation [147]. Additionally, miR-3436 negatively regulates YTHDF1 in human glioblastoma [148]. The expression of YTHDF2 is significantly upregulated in epithelial ovarian cancer tissues, and the upregulation of YTHDF2 expression reduces the overall m6A-mRNA levels. miR-145 directly targets and downregulates YTHDF2 levels, indirectly upregulating m6A levels, thereby inhibiting the proliferation and migration of epithelial ovarian cancer cells [149]. miR-6125 targets YTHDF2 to downregulate YTHDF2 protein expression, and this increases the stability of glycogen synthase kinase 3β(GSK3β) mRNA via m6A modification. Elevated GSK3β protein levels can inhibit the expression of proteins related to the Wnt/β-catenin/Cyclin D1 pathway, leading to G0-G1 phase arrest and ultimately inhibiting the proliferation of CRC cells [150]. Additionally, in prostate cancer, miR-493-3p directly targets and reduces YTHDF2 levels to increase downstream m6A levels, thereby inhibiting the proliferation and migration of prostate cancer cells [151]. YTHDF2, as a target of miR-495, induces mRNA degradation by recognizing the m6A modification of MOB kinase activator 3B (MOB3B) mRNA, thereby inhibiting the expression of MOB3B [152].

### 4.2. lncRNA/circRNA

lncRNA is a transcript longer than 200 nucleotides without protein-coding function [153]. CircRNA lacks 3′ and 5′ ends, usually generated by back-splicing of precursor mRNA, and exists in a circular form [153]. Both lncRNA and circRNA regulate gene expression through various mechanisms. They can act as miRNA sponges to reduce miRNA levels and prevent the degradation of target mRNA. They can also regulate the binding of transcription factors to promoters, thereby modulating the expression of target genes [154].

The inhibitory role of circRNAs in tumor progression is manifested through their interactions with members of the IGF2BPs family. Xie et al. focused on the oncogenic function of IGF2BP1 in breast cancer progression and identified two novel circRNAs with potential binding ability to IGF2BP1. Further research confirmed that circPTPRA, derived from the pre-mRNA of the PTPRA protein, interacts with IGF2BP1. CircPTPRA competitively binds to the KH domain of IGF2BP1, interfering with its interaction with the downstream targets Myc and FSCN1 mRNA, thus inhibiting the growth and invasiveness of breast cancer cells [155]. In clear cell renal cell carcinoma (ccRCC), a circRNA from the transportin 3 (TNPO3) gene, circ-TNPO3, directly binds to the IGF2BP2 protein, destabilizing SERPINH1 mRNA and inhibiting ccRCC cell proliferation [156]. Previous studies have suggested that circ-TNPO3 acts as a protein decoy to inhibit migration in GC [157]. Intensive studies conducted on circ-TNPO3 have proven that it is a novel tumor suppressor. However, studies show that circTNPO3 acts as a sponge for miR-1299, promoting resistance to paclitaxel in OC cells through the circTNPO3/miR-1299/NEK2 signaling pathway [158]. This necessitates cautious consideration of circTNPO3 as an anticancer agent. Regarding the inhibitory effect of circRNA on IGF2BP3, it is exemplified by circRNA CDR1as in melanoma cell metastasis [159].

A novel circRNA, circFAT1(e2), is expressed in both the cytoplasm and nucleus of GC cells and exerts anticancer effects through different pathways. In the cytoplasm, circFAT1(e2) overexpression acts as a sponge to downregulate miR-548g, upregulating RUNX1 expression and significantly reducing GC proliferation, invasion, and migration. In the nucleus, circFAT1(e2) directly targets YBX-1 to inhibit GC growth [160]. Additionally, cFAM210A binds to YBX-1 and inhibits its phosphorylation, suppressing its transactivation function on the Mesenchymal-Epithelial Transition Factor to inhibit HCC occurrence [161]. CircNEIL3 recruits E3 ubiquitin ligase Nedd4L to degrade YBX-1, inhibiting tumor metastasis [162].

The lncRNA LINC00472 can inhibit the EMT process in lung adenocarcinoma cells by binding to YBX-1. Notably, besides its anticancer function, the association of LINC00472 with the mechanical properties of lung adenocarcinoma cells represents the first report on the relationship between lncRNA and cancer cell mechanical properties [163]. The host gene of miR-503, lncRNA miR503HG, significantly inhibits the invasion and metastasis of liver cancer. Mechanistically, miR503HG specifically binds to HNRNPA2B1 and promotes its degradation through the ubiquitin-proteasome pathway, thereby reducing the stability of p52 and p65 mRNA and inhibiting the NF-κB signaling pathway. Additionally, the study found that miR503HG can synergistically interact with miR503 to inhibit HCC proliferation and migration [164].

LncRNA FGF13-AS1 disrupts the interaction between IGF2BP1 and Myc mRNA through the FGF13-AS1/IGF2BP1/Myc axis, inhibiting glycolysis and stemness of breast cancer cells. Furthermore, Myc negative feedback inhibits FGF13-AS1 transcription, forming an overall feedback loop [165]. LINC01093 directly targets IGF2BP1, disrupting its interaction with glioma-associated oncogene homolog 1 mRNA and further affecting the expression of downstream molecules involved in HCC progression [166]. However, as exemplified by YBX-1, the interaction modes and outcomes between lncRNAs and readers in cancer are diverse [167,168]. Some lncRNAs have low targeting specificity, which could potentially have detrimental effects in normal cells. The specific mechanistic differences need further validation. Therefore, there is still a long way to go to better utilize them as inhibitors.

### 4.3. siRNA Nano-Carrier Delivery

Distinguishing from naturally occurring ncRNAs, small interfering RNA (siRNA) is derived from artificially constructed double-stranded RNA cleavage products [169]. It exhibits potent post-transcriptional gene-silencing effects with strong specificity and targeting. It has been demonstrated that complementary siRNA downregulation of reader genes is an effective anticancer method [170]. However, siRNA faces numerous limitations in vivo, such as elimination, immune destruction, instability, toxicity, and off-target effects [171]. To enhance siRNA functionality, nanocarriers such as liposomes have been developed for encapsulation to adapt to the internal biological environment, maximizing stability to reach targets and effectively silence genes of interest [172]. Rapid development in this field will undoubtedly advance the progress of reader inhibitors simultaneously.

Transferrin (Tf), a serum protein that binds iron, exhibits a specific affinity for transferrin receptors (TfRs) on the cell membrane [173]. Lung cancer cells often overexpress TfR [174], making them a potential mediator for delivering anticancer drugs. Based on this, Muralidharan et al. utilized Tf as a targeting ligand to modify chemically synthesized DOTAP:Chol nanoparticles encapsulating HuR siRNA, creating a targeted delivery system for tumor-specific nanoparticle delivery of HuR siRNA. In this therapy, effective knockdown of HuR levels and its associated protein expression by HuR siRNA resulted in superior tumor size reduction compared to the control siRNA group. Importantly, this therapy had no significant effect on normal lung fibroblast cells, demonstrating selective activity against lung cancer cells while being safe for normal cells [175]. Similarly, folate receptor-α (FRA) is overexpressed in lung cancer cells, allowing folic acid to be conjugated to nanoparticles encapsulating HuR siRNA for targeted delivery of FRA and accurate release of HuR siRNA. However, a single knockdown of HuR is insufficient to eliminate cancer completely.

Cis-diamine platinum (CDDP), a platinum-based anticancer drug, is commonly used to treat lung cancer [176]. Its main limitation is its function in normal tissues, which leads to nonspecific toxicity in normal cells. Taking advantage of these features, Amreddy et al. developed a nanoparticle system based on folate-conjugated dendrimeric polyamidoamine (Den) for the co-delivery of HuR siRNA and CDDP to FRA-overexpressing lung cancer cells. Co-encapsulation of siRNA and chemotherapy drugs improved therapeutic efficacy while reducing cytotoxicity to normal cells [177]. Mesenchymal stem cell (MSC)-derived small extracellular vesicles (MsEVs) tend to be recruited to tumor sites and participate in tumor progression, a phenomenon known as the tumor homing effect, making them suitable drug delivery systems for cancer treatment [178]. Encapsulation of YTHDF1 siRNA and docetaxel (DTX), a first-line chemotherapy drug for ovarian cancer, into MSC-derived MsEVs, resulted in significant tumor targeting and internal/lysosomal escape of YTHDF1 siRNA. It effectively knocked down YTHDF1 and significantly enhanced the anti-OC effects of DTX [179]. Additionally, targeting YTHDF1 using LNP-encapsulated YTHDF1 siRNA also contributed to inhibiting the progression of HCC [180]. With advancements in materials science, the design of more compatible and high-performance encapsulation systems is expected to be explored further.

## 5. Small-Molecule Inhibitors of Readers

The azopodophyllotoxin small molecule SU056 is an effective YBX-1-targeting inhibitor. In vivo, SU056 targets YBX-1 to promote apoptosis and RNA degradation pathway protein enrichment while simultaneously downregulating splicing pathways to independently inhibit OC progression. In combination with paclitaxel without significant hepatotoxicity, it further reduces disease progression [181]. The targeted inhibitory effect of SU056 on YBX-1 has been confirmed in acute myeloid leukemia (AML) [182], lung cancer [183], and breast cancer [184].

Pyrvinium pamoate, an FDA-approved anthelmintic drug, is a novel HuR inhibitor. It dose-dependently inhibits HuR accumulation in the cytoplasm by activating the AMP-activated protein kinase/importin α1 cascade, promoting HuR nuclear import, and blocking HuR nuclear-cytoplasmic translocation via checkpoint kinase 1/tumor necrosis factor-α receptor-associated factor 1 pathway inhibition. Combining pyrvinium pamoate with chemotherapy drugs inhibits the growth of mouse bladder tumor xenografts [185]. MS-444 is a small-molecule inhibitor of HuR that disrupts HuR cytoplasmic transport, releasing COX-2 and other ARE-mRNAs, targeting them to P-bodies, and inhibiting their expression levels. MS-444 is well tolerated and, after intraperitoneal administration in mice, inhibits CRC tumor growth by enhancing apoptosis and reducing angiogenesis [186]. Cryptotanshinone, a major lipophilic component isolated from Danshen (*Salvia miltiorrhiza* Bunge), also inhibits HuR nuclear-cytoplasmic translocation, destabilizing TNF-α mRNA, thereby inhibiting the proliferation of melanoma cells [187]. Similarly, small-molecule inhibitors that achieve cancer suppression by inhibiting HuR nuclear-cytoplasmic transport include YM-155 [188], MPT0B098 [189], SP600125 [190], AZA and TSA [191], N-benzylcantharidinamide [192], triptolide [193], latrunculin A [194], blebbistatin [194], which are effective in chronic myeloid leukemia [188], lung adenocarcinoma [189], breast cancer [190,191], HCC [192,194], and NSCLC [193].

Traditional anti-inflammatory Chinese medicine 15,16-Dihydrotanshinone-I (DHTS) prevents HuR:RNA complex formation. Specifically, DHTS interacts with HuR, stabilizing it in a locked conformation, hindering competitive RNA binding, and inhibiting the migration of breast cancer, cervical adenocarcinoma, and colon carcinoma cells [195,196]. CMLD-2 is a promising HuR-targeting therapeutic drug that selectively reduces HuR and HuR-regulated protein (Bcl2 and p27) mRNA levels in tumor cells. Compared to normal cells, CMLD-2 treatment causes mitochondrial disruption, Caspase-9 and -3 activation, and PARP cleavage in NSCLC cells [197]. Similarly, small molecules that have been identified to inhibit the binding of HuR to target mRNA by competitively targeting the HuR-binding site can also be grouped together. Similar small molecules include mitoxantrone [198], suramin [199], KH3 [200], and eltrombopag [201], and their efficacy in cancer types is presented in Table 1.

Small-molecule inhibitors of HNRNPA1 are numerous, with compound VPC-80051 as the first small-molecule inhibitor of HNRNPA1 splicing activity discovered through computer-aided drug design. It interacts directly with HNRNPA1 RBD, lowering downstream AR-V7 levels, thus inhibiting castration-resistant prostate cancer [202]. Riluzole, a drug for treating amyotrophic lateral sclerosis, is an inhibitor of HNRNPA1. Riluzole binds directly to HNRNPA1 and inhibits IRES activity by affecting ITAF/RNA binding. Riluzole also exhibits synergistic effects with mTOR inhibitors against glioblastoma multiforme [203]. Another unnamed compound, 11, achieves glioblastoma inhibition by blocking HNRNPA1 interaction with IRES [204]. The natural polyphenolic compounds quercetin, esculetin, and tetracaine hydrochloride exert effects on prostate cancer, endometrial cancer, and melanoma by disrupting HNRNPA1 nuclear-cytoplasmic transport [205,206,207]. Computer-aided protein chemistry identified the pseudo-uric derivative XI-011, which binds to the RRM1 domain of hnRNPA2B1, disrupting HnRNPA2B1/nucleic acid interactions and inhibiting the MDMX-p53 axis in GC. This method provides a new strategy for treating GC by chemically targeting hnRNPA2B1 to restore p53 activity [208].

The small molecule BTYNB is a potent, selective inhibitor of IGF2BP1 binding to c-Myc mRNA. BTYNB downregulates β-TrCP1 mRNA, reducing activation of nuclear transcription factor- κB. eEF2, an oncogenic translational regulator, becomes a new IGF2BP1 target mRNA, allowing BTYNB to effectively inhibit the proliferation of OC and melanoma cells containing IGF2BP1 [209]. Small molecule 7773 interacts with the hydrophobic surface bordering the KH3 and KH4 domains of IGF2BP1, inhibiting its binding to Kras RNA and improving lung cancer progression [210]. Lapatinib inhibits the binding of IGF2BP2 to ErbB2 mRNA, overcoming resistance to tyrosine kinase inhibitors, a major obstacle in the treatment of radioactive iodine-refractory papillary thyroid carcinoma (RR-PTC) [211]. Dahlem et al. validated 10 compounds belonging to the benzofuran amino benzene carboxylic acid and thiazole classes, showing in vitro specificity for IGF2BP2. The three most potent compounds inhibit the growth of CRC and liver cancer in vivo [212]. Additionally, the small-molecule IGF2BP2 inhibitors JX5 and CWI1-2 exhibit good efficacy against leukemia [213,214]. The derivative of isocorydine (d-ICD) inhibits IGF2BP3 expression in a time-dependent manner, inhibits HCC cell growth, and reduces sorafenib resistance in HCC cells [215]. Bromodomain and extra-terminal domain inhibitors (BETi), such as JQ1, reduce IGF2BP3 expression, alter the expression of its validated targets, and inhibit Ewing sarcoma cell growth under anchorage-dependent conditions [216].

Gallotannin acetate (GAA) is the first specific LRPPRC inhibitor. In P53-mutated CRC cells, GAA effectively induces LRPPRC protein degradation, reducing CRC therapy resistance. Furthermore, combined chemotherapy with GAA and 5-fluorouracil improves CRC treatment outcomes [145]. As an effective drug, GAA-induced LRPPRC degradation also inhibits lung adenocarcinoma and OC development [217,218]. Tegaserod, an FDA-approved drug, is a YTHDF1 inhibitor that blocks YTHDF1 from directly binding to m6A-modified mRNA, inhibiting leukemia occurrence [219].

Apart from traditional methods, computer-aided drug design (CADD) is widely used for screening inhibitors of drug targets. This undoubtedly contributes to further developments in the discovery process of targeted drugs, including predicting the interactions between compounds and targets using computational methods and optimizing the structures of drug candidates [220]. Of course, the predicted results need experimental validation to ensure their efficacy and safety in living organisms. Molecular dynamics simulations and nuclear magnetic resonance spectroscopy are commonly used to validate the CADD results. In vitro experiments utilize high-throughput screening methods for compound screening, with the fluorescence polarization assay being one of the most commonly used techniques. Other validation methods include pull-down assays and enzyme-linked immunosorbent assays. Further validation can be performed using cellular methods for protein-protein interactions, such as fluorescence resonance energy transfer and proximity ligation assays [221]. Through a comprehensive evaluation of these methods, hundreds of inhibitors targeting RNA readers have been screened through the collaborative efforts of many groups (as shown in Table 1) [222,223,224,225], providing more selective choices for subsequent experiments. However, these inhibitors still require further in vitro and in vivo validation to clarify their targeting of cancer types and efficacy levels. The use of computer-assisted development in the field of targeted drugs will continue to unleash their enormous potential.

**Table 1:** Small-molecule inhibitors of readers in cancer.

Readers	Inhibitor	Cancer Type	Inhibition Mechanism	Ref.
ALYREF	CHEMBL3752986; CHEMBL3753744	neuroblastoma	Directs targeting of ALYREF protein	[226]
YBX-1	SU056	AML	May interfere with the binding of oncogenic mRNAs to YBX-1	[182]
		OC	May interfere with the binding of oncogenic mRNAs to YBX-1	[181]
		breast cancer	May interfere with the binding of oncogenic mRNAs to YBX-1	[183]
		lung cancer	May interfere with the binding of oncogenic mRNAs to YBX-1	[184]
	F2,3;C1,2,3,6,8,11,12;A3;P1	Not Determined	Interferes with the binding of mRNA to YBX-1 in cells	[223]
HuR	pyrvinium pamoate	UCB	Inhibits the cytoplasmic translocation of HuR	[185]
	MS-444	CRC	Inhibits the cytoplasmic translocation of HuR	[186]
	YM-155	CML	Inhibits the cytoplasmic translocation of HuR	[188]
	Cryptotanshinone	Melanoma	Inhibits the cytoplasmic translocation of HuR	[187]
	MPT0B098	Lung cancer	Inhibits the cytoplasmic translocation of HuR	[189]
	Dehydromutactin	Not Determined	Inhibits the cytoplasmic translocation of HuR	[224]
	Okicenone	Not Determined	Inhibits the cytoplasmic translocation of HuR	[224]
	JNK inhibitor (SP600125)	Breast cancer	Inhibits the cytoplasmic translocation of HuR	[190]
	AZA and TSA	Breast cancer	Inhibits the cytoplasmic translocation of HuR	[191]
	NBenzylcantharidinamide	HCC	Inhibits the cytoplasmic translocation of HuR	[192]
	Triptolide	NSCLC	Inhibits the cytoplasmic translocation of HuR	[193]
	Leptomycin B	Not Determined	Inhibits the cytoplasmic translocation of HuR	[227]
	Latrunculin A	HCC	Inhibits the cytoplasmic translocation of HuR	[194]
	Blebbistatin	HCC	Inhibits the cytoplasmic translocation of HuR	[194]
	DHTS	Breast cancer	Inhibits HuR binding to target mRNAs	[195]
		Cervical cancer; colon cancer	Inhibits HuR binding to target mRNAs	[196]
	AZA-9	Not Determined	Inhibits HuR binding to target mRNAs	[228]
	Mitoxantrone	Breast cancer	Inhibits HuR binding to target mRNAs	[198]
	Suramin	Oral cancer	Inhibits HuR binding to target mRNAs	[199]
	C10,11	Not Determined	Inhibits HuR binding to target mRNAs	[229]
	CMLD1,3,4,5,6	Not Determined	Inhibits HuR binding to target mRNAs	[225]
	CMLD2	NSCLC	Inhibits HuR binding to target mRNAs	[197]
	Quercetin	TNBC	Inhibits HuR binding to target mRNAs	[230]
	KH3	Breast cancer	Inhibits HuR binding to target mRNAs	[200]
	compound **5,7,2**	Not Determined	Interferes with HuR–RNA binding	[231]
	Tanshinones 6b-6i,6k-6t,6w	Not Determined	Interferes with HuR–RNA Interaction	[232]
	STK018404	Not Determined	Targets HuR	[233]
	compound **2,3**	Not Determined	Interferes with the HuR–RNA complex	[234]
	eltrombopag	breast cancer	Interferes with the HuR–RNA complex	[201]
	compound **1,3,4**	Not Determined	Targets HuR	[235]
HNRNPA1	VPC-80051	CRPC	Targets the RNA-binding domain (RBD) of HNRNPA1	[202]
	Camptothecin	Not Determined	Binds to HNRNPA1 and inhibits the HNRNPA1/ topoisomerase I (top I) interaction	[236]
	Riluzole	Glioblastoma	Binds to HNRNPA1 and inhibits IRES activity via effects on ITAF/RNA binding	[203]
	Compound **11**	Glioblastoma	Blocks HNRNPA1 from interacting with IRES of c-Myc and cyclin D1	[204]
	Quercetin	Prostate Cancer	Binds to and impairs the ability of HNRNPA1 to shuttle between the nucleus and cytoplasm, resulting in its cytoplasmic retention	[205]
	Tetracaine hydrochloride	melanoma	translocation of HNRNPA1 from the nucleoplasm to the nuclear envelope and reduced the protein stability of HNRNPA1	[237]
	AR-A 014418	Glioblastoma	Inhibits of GSK3 kinase leads to downregulation of HNRNPA1	[238]
	Esculetin	endometrial cancer	binds directly to the HNRNPA1 protein, affecting the export of the HNRNPA1/mRNA complex from the nucleus into the cytoplasm	[206]
HNRNPA2B1	VPC-80051	Not Determined	inhibits mRNA translation by binding to the C-terminal glycine-rich domain of hnRNPA2B1	[239]
	Camptothecin	Gastric carcinoma	disrupts HnRNPA2B1/nucleic acid interactions	[208]
	Riluzole	Not Determined	Targeting the cyclophilin domain of Ran-binding protein 2 (Ranbp2) to indirectly downregulate the proteostasis of hnRNPA2B1	[240]
IGF2BP1	BTYNB	Melanoma; OC	a potent and selective inhibitor of IMP1 binding to c-Myc mRNA	[209]
	7773	lung cancer	interacts with a hydrophobic surface at the boundary of Igf2bp1 KH3 and KH4 domains, and inhibits binding to Kras RNA	[210]
IGF2BP2	JX5	T-ALL	bind IGF2BP2 KH3-4 domains	[213]
	CWI1-2	AML	preferentially binds to IGF2BP2 and inhibits its interaction withm6A-modified target transcripts	[214]
	lapatinib	RR-PTC	Inhibition of IGF2BP2 binding to ErbB2 mRNA	[211]
	compound **1–10**	Colorectal; liver cancer	Target specificity of IGF2BP2	[212]
IGF2BP3	d-ICD(8-Amino-isocorydine)	HCC	/	[215]
	I-BET151	MLL	/	[241]
	BETi JQ1	Ewing Sarcoma Malignancy	/	[216]
LRPPRC	GAA	CRC	binds to LRPPRC directly, disrupts the interaction of LRPPRC with its stabilizing chaperon protein, and leads to LRPPRC degradation by mitochondrial protease	[145]
		lung cancer	binds to LRPPRC directly, disrupts the interaction of LRPPRC with its stabilizing chaperon protein, and leads to LRPPRC degradation by mitochondrial protease	[217]
		OC	binds to LRPPRC directly, disrupts the interaction of LRPPRC with its stabilizing chaperon protein, and leads to LRPPRC degradation by mitochondrial protease	[218]
YTHDC1	Fragment 1–30	Not Determined	disrupts YTHDC1-RNA interactions	[242]
	Compound **1–27**	Not Determined	disrupts the interactions between mRNA and YTHDC1	[243]
	compound **40**	AML	selectively targets YTHDC1	[244]
	YL-5092	AML	selectively targets YTHDC1	[245]
YTHDF1	salvianolic acid(SAC)	Not Determined	Disrupts the binding of YTHDF1 to its substrate RNA, SAC dissolves YTHDF1 condensates and counteracts hyperactive YTHDF1 in neurons	[246]
	tegaserod	AML	blocks the direct binding of YTHDF1 with m6A-modified mRNAs	[219]
	compound **1–10**	Not Determined	disrupts the interaction of the YTHDF m6A domain with the m6A-decorated mRNA targets	[247]
YTHDF2	compound **1–17**	Not Determined	competes with m6A for binding to the m6A-reader domain of YTHDF2	[248]
	Fragment 26	Not Determined	competes with m6A for binding to the m6A-reader domain of YTHDF2	[249]

## 6. Other Inhibitors of Readers

In addition to ncRNAs and small molecules serving as reader inhibitors, other molecules such as RNA, proteins, and enzymes can also exert equivalent inhibitory effects on readers. Importin α1 is an adaptor protein involved in nuclear import and promotes the nuclear import of HuR through two AMPK regulatory mechanisms. However, importin α1 proteins harboring K22R or S105A mutations lose this ability, so mutant importin α1 inhibits the progression of CRC cells by suppressing cytoplasmic translocation of HuR [250]. S-adenosylmethionine, a coenzyme, also inhibits the cytoplasmic translocation of HuR and suppresses the cell cycle progression of HCC cells by blocking AMPK phosphorylation [251]. Sun et al. indicated that some small functional motifs or elements in eukaryotic mRNA may still function in vivo after separation from their parent molecules. A 62nt AU-rich RNA from C/EBPb 3′UTR, R62, which competes specifically with C/EBPb mRNA to bind to HuR, inhibits the proliferation of HCC cells [252].

BC15 is an HNRNPA1-specific single-stranded DNA aptamer whose inhibitory effect on liver cancer cell proliferation is even stronger than that of HNRNPA1 small interfering RNA, suggesting its potential as a candidate inhibitor of HNRNPA1 [253]. Enterovirus 71 (EV71) released 3C protease can induce cell apoptosis and promote the release of virus particles. Mechanistically, EV71 infection or ectopic expression of 3C protease can cleave HNRNPA1, disrupting the HNRNPA1-apaf-1-caspase-3-apoptosis axis, ultimately leading to cell lysis and virus release [254]. E3 ubiquitin ligase zinc finger protein 91 promotes Lys48-linked ubiquitination and proteasomal degradation of HNRNPA1 at Lys8, inhibiting HNRNPA1-dependent PKM alternative splicing, shifting toward higher PKM1 and lower PKM2 subtypes, and suppressing HCC glucose metabolism reprogramming, cell proliferation, and metastasis [255].

Deubiquitinase ubiquitin-specific protease 47 prevents ubiquitination of YTHDF1 and weakens its association with the translation initiation machinery, thereby reducing the translation efficiency of c-Myc based on m6A [256]. F-box and WD repeat domain-containing 7, an important component of the E3 ubiquitin ligase complex, counteracts the oncogenic role of YTHDF2 by inducing the proteasomal degradation of YTHDF2 in OC [257].

## 7. Conclusions and Perspective

In this review, we outline the multifaceted roles of RNA readers, elucidate their oncogenic mechanisms in cancer, and focus on the comprehensive examination of ncRNA, small molecules, and alternative inhibitors. Each inhibitor category presents unique advantages and disadvantages: ncRNA inhibitors are of high specificity to the targeted RNA sequences and thus have relatively lower off-target effects compared to small-molecule inhibitors, which usually cause side effects due to their small size and potential to interact with multiple proteins. ncRNAs offer precise targeting capabilities with minimal off-target effects; small molecules boast favorable pharmacokinetic properties conducive to drug development, and the relatively simple chemical structures of small-molecule compounds facilitate high-throughput screening to identify potent inhibitors targeting RNA readers, while the development and optimization of ncRNA inhibitors can be quite complex due to the limited understanding of their biological functions and mechanisms. The synthesis of non-coding RNA inhibitors usually involves specialized techniques and facilities, which are complex and expensive, while small-molecule synthesis (with some exceptions) is rather straightforward and less expensive due to the extensive knowledge base and established synthetic routes. The delivery of ncRNA inhibitors to the target cells or tissues can be challenging, and they often need to be protected from degradation and thus to be transported across cell membranes, while small-molecule inhibitors generally have better cell permeability, and their deliveries are relatively easy. RNA molecules are often unstable compared to small molecules and are susceptible to degradation by enzymes such as nucleases. Other types of inhibitors provide diverse mechanisms for interference, allowing for complementary approaches.

Prospects stemming from the utilization of inhibitors of RNA readers are profoundly encouraging. Inhibitors targeting RNA readers possess the ability to modulate critical signaling pathways in cancer cells, thereby inhibiting cell proliferation, promoting apoptosis, and exerting other anticancer effects. Furthermore, the combination of inhibitors with chemotherapy drugs can effectively suppress signaling pathways associated with drug resistance in cancer cells, thereby reducing cellular defense mechanisms against chemotherapy agents [258]. This sensitizes cancer cells and amplifies the cytotoxic effects of chemotherapy drugs. For instance, co-administration of miR-137 with the chemotherapy drug OXA sensitizes GC cells to OXA [119]. Similarly, combination chemotherapy utilizing gemcitabine and 5FU has demonstrated improved treatment outcomes in CRC [145]. Future investigations of inhibitors may contemplate these dimensions. Additionally, considering the co-encapsulation of inhibitors with chemotherapeutic drugs seems to overcome the stability protection and drug release rate issues for both, which also represents a direction for future development [177].

In an article published by Megan Cully in News and Analysis in 2019, it was mentioned that FDA-approved drugs targeting histones already exist. The author also discussed that at the inaugural conference on RNA epigenetics held in Cambridge, UK, some companies had put clinical trials for RNA modification enzyme inhibitors on their agendas. For instance, Gotham Therapeutics aimed to initiate clinical trials for their METTL3 inhibitor in 2022, marking a historic breakthrough in RNA epigenetics [259]. Although there are currently no approved drugs targeting readers, we know that given the rising interest in this field in recent years, such developments will occur in the near future.

## Abbreviations

m5C:5-Methylcytosine; m6A:N6-Methyladenosine; HNRNPs: Heterogeneous nuclear ribonucleoproteins; IGF2BPs:Insulin-like growth factor 2 mRNA-binding proteins; HuR: human antigen R; HNRNPA1: Heterogeneous Nuclear Ribonucleoprotein A1; m6Am: N6-methyladenosine with 2′-O-methylation; ALYREF: Aly/REF export factor; YBX-1: Y-box binding protein 1; TREX: transcription export complex; RRMs: RNA recognition motifs; FMRP: fragile X mental retardation protein; hnRNA: heterogeneous nuclear RNA; IRES: internal ribosome entry sites; lncRNA: long non-coding RNA; circRNAs: circular RNAs; SRSF; Serine/arginine-rich splicing factors; LRPPRC: Leucine-rich pentatricopeptide repeat-containing protein; PRRC2A: Proline-rich coiled-coil 2A; 5′-UTR: 5′-untranslated region; DCP2: decapping mRNA 2; HCC: hepatocellular Carcinoma; UCB: urothelial bladder carcinoma; OC: ovarian cancer; CRC: colorectal cancer; GC: gastric cancer; EMT: epithelial-to-mesenchymal transition; AREs: AU-rich elements; ncRNA: Non-coding RNA; miRNA: microRNA; circRNA: circular RNA; ECs: Endothelial cells; EVs: extracellular vesicles; TNPO3: transportin 3; siRNA: small interfering RNA; CDDP: cis-diamine platinum; AML: acute myeloid leukemia; DHTS: 15,16-Dihydrotanshinone-I; GAA: Gallotannin acetate.
